# PPARG agonist pioglitazone influences diurnal kidney medulla mRNA expression of core clock, inflammation‐, and metabolism‐related genes disrupted by reverse feeding in mice

**DOI:** 10.14814/phy2.15535

**Published:** 2022-12-13

**Authors:** Olga Izmailova, Alina Kabaliei, Viktoriya Shynkevych, Oksana Shlykova, Igor Kaidashev

**Affiliations:** ^1^ Poltava State Medical University Poltava Ukraine

**Keywords:** clock genes, kidney, pioglitazone, PPARG, reverse feeding

## Abstract

This study examined the influence of PPARG activation by pioglitazone (PG) on the mRNA of core clock, inflammation‐ and metabolism‐related genes in the mouse kidney medulla as well as urinary sodium/potassium excretion rhythms disrupted by reverse feeding. Mice were assigned to daytime feeding and nighttime feeding groups. PG 20 mg/kg was administered at 7 am or 7 pm. On day 8 of the feeding intervention, mice were killed at noon and midnight. Kidney medulla expression of *Arntl, Clock, Nr1d1, Cry1, Cry2, Per1, Per2, Nfe2l2, Pparg*, and *Scnn1g* was determined by qRT PCR. We measured urinary K^+^, Na^+^, urine volume, food, and H_2_O intake. The reverse feeding uncoupled the peripheral clock gene rhythm in mouse kidney tissues. It was accompanied by a decreased expression of *Nfe2l2* and *Pparg* as well as an increased expression of *Rela* and *Scnn1g.* These changes in gene expressions concurred with an increase in urinary Na^+^, K^+^, water excretion, microcirculation disorders, and cell loss, especially in distal tubules. PG induced the restoration of diurnal core clock gene expression as well as *Nfe2l2, Pparg*, *Scnn1g* mRNA, and decreased *Rela* expressions, stimulating Na^+^ reabsorption and inhibiting K^+^ excretion. PG intake at 7 pm was more effective than at 7 am.

## Graphical abstract

This study examined the influence of PPARG activation by pioglitazone (PG) on the mRNA of core clock, inflammation‐, and metabolism‐related genes in the mouse kidney medulla as well as urinary sodium/potassium excretion rhythms disrupted by reverse feeding. The reverse feeding induced disruption of mouse kidney diurnal expression pattern of core clock genes, increasing *Rela* and *Scnn1g* expression and urinary Na^+^, K^+^, water excretion, decreasing *Nfe2l2* and Pparg expression. PG restores the diurnal core clock gene expression pattern, decreases *Rela* and *Scnn1g* expression, increases *Nfe2l2* and *Pparg* mRNA, stimulates Na^+^ reabsorption, and inhibits K^+^ excretion.

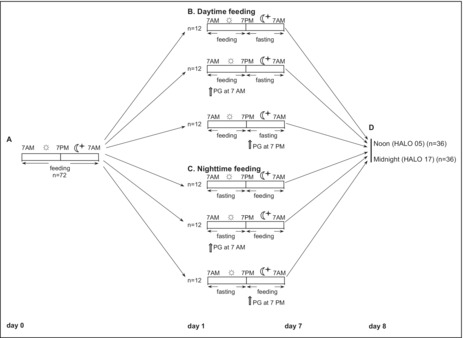

## 1 INTRODUCTION

Renal function is essential for maintaining systemic homeostasis by blood filtration, water and electrolyte balance, and regulation of blood pressure. These processes have clear rhythmicity—urinary sodium, potassium, chloride as well as urine volume display a 24‐h rhythmic pattern (Mills & Stanbury, 1952). The rhythmicity of renal functions depends on the central clock, localized in the suprachiasmatic nucleus of the hypothalamus, and the peripheral clocks, involving other organs such as the kidneys (Sehgal, 2017).

The peripheral clock comprises transcription factors ARNTL and CLOCK, which bind response elements in the promoters of target genes—*Per, Cry*, etc. This mechanism contributes to the regulation of nearly half of all expressed genes in a tissue‐specific manner (Takahashi, 2017). In the kidney, the clock mechanism regulates xenobiotic metabolism and various sodium transport genes such as Na^+^–Cl^−^ cotransporter and prostasin (Castagna et al., 2015; Douma et al., 2018; Nikolaeva et al., 2016; Stow et al., 2012).

Modern lifestyle leads to the disruption of the circadian system due to misalignment between the endogenous circadian rhythms of internal body clocks and the external environment (Pilorz et al., 2018). Moreover, feeding and light cycle affect a large number of key kidney physiological functions, such as renal plasma flow and urine production (Gachon et al., 2004; Wu et al., 2012, 2010). In shift workers and employees with long working hours, such disruption increased the risk of developing chronic kidney disease and was associated with declining renal function (Lee et al., 2020; Uhm et al., 2018). At present, we have growing evidence that loss of circadian rhythm is observed in patients with renal disorders such as chronic kidney disease, lupus nephritis, and age‐related renal structural changes (Mohandas et al., 2022). Additional studies have shown that impaired circadian rhythm can be seen prior to further renal damage in patients with either type 1 or type 2 diabetes (Felício et al., 2010; Lurbe et al., 2002; Vörös et al., 1998). Experimental diabetes induction induced a disbalance in core clock gene expression in the mouse kidneys—an increase in the level of *Per1* and a decrease in the levels of *Per2* (Oishi et al., 2004).

Core clock genes have direct interactions with PPARG (Kiss‐Tóth & Roszer, 2008; Sarafidis & Lasaridis, 2006; Yang et al., 1999), suggesting that it may act as a molecular link between circadian rhythm and energy metabolism. For example, *Per2* represses PPARG by blocking its recruiting to target promoters, a mechanism that is conceptually different from the repression that *Per2:Cry* exerts on *Clock:Arntl* (Bae et al., 2001). From the other hand, *Nr1d1*, one of the core clock components, is the target gene of PPARG which binds to the direct repeat (DR)‐2 response element Rev‐DR2 (Fontaine et al., 2003). Deletion of PPARG in mice suppresses or diminishes diurnal rhythms (Yang et al., 2012).

In the kidney, PPARG is expressed in different regions of the renal collecting system under physiological conditions, including connecting renal tubules and collecting ducts (Yang et al., 1999). PPARG is also abundant in the inner renal medulla and is localized to the epithelial layer, from the medullary collecting ducts to the urothelium of the ureter and the bladder. It is additionally expressed in renal medullary interstitial cells, the juxtaglomerular apparatus, and the glomeruli, including podocytes, mesangial cells, and renal microvascular endothelial cells (Kiss‐Tóth & Roszer, 2008). Given that multiple renal cell types have endogenous PPARG expression and activity, its activation in the kidney may be critical for governing renal function.

Synthetic PPARG ligand agonists have been shown to have renoprotective effects both in diabetic and nondiabetic patients (Sarafidis & Bakris, 2006). However, up to 5% of patients experience the treatment‐limiting side effect of edema and body weight increase (Vasudevan & Balasubramanyam, 2004). Recent observation and the density of expression of PPARG in the proximal tubule suggest that Na^+^ and water retention may be due to PPAR stimulation of proximal tubular Na^+^ transporters (Berlie et al., 2007). From the other side, there are conflicting reports concerning the influence of PPARG agonists on fluid retention. PPARG agonists stimulated epithelial Na^+^ channel (ENaC) activity (Chraïbi & Renauld, 2014; Renauld et al., 2010). Researchers reported that PPARG agonists decreased Na^+^ transport via ENaC (Pavlov et al., 2009). Other studies found that the significant increases in the renal expression of the ENaC subunits α, β, or γ (encoded by genes *Scnn1a, Scnn1b*, and *Scnn1g*) could not be detected in response to PPARG agonists (Chen et al., 2005; Song et al., 2004; Vallon et al., 2009). Borsting E. et al. (2012) showed that PPARG agonists did not increase ENaC mRNA expression in the kidney, but repressed the *Scnn1g* promoter via an indirect transcriptional mechanism (Borsting et al., 2012). Thus, there are a lot of controversies in the question how PPARG agonists influence *Scnn1g*.

Hogenesch et al. show that PPARG does have a circadian rhythm in the kidney (Pizarro et al., 2013). An evaluation of this phenomenon could be especially interesting to associate the regulation of renal metabolism with central and peripheral circadian networks (Corrales et al., 2018). In our previous study, we demonstrated that PPARG agonist pioglitazone (PG) influenced mouse liver mRNA expression of clock genes and inflammation‐related genes disrupted by reverse feeding (Fedchenko et al., 2022). This data supposed extrahepatic effects of PG and their influence on metabolism in this model.

This study examined the influence of PG on the diurnal mRNA expression profile of core clock genes, inflammation‐ and metabolism‐related genes in the mouse kidney medulla as well as diurnal urinary sodium and potassium excretion rhythms disrupted by reverse feeding.

## 2 MATERIALS AND METHODS

### 2.1 Animals

**FIGURE 1:**
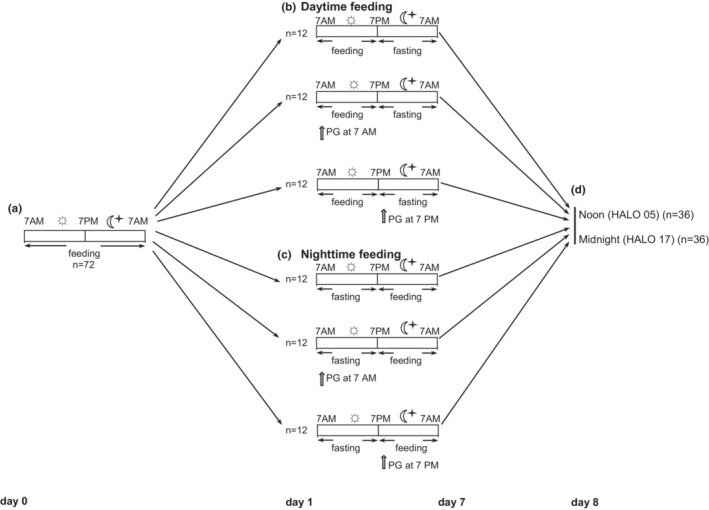
Experimental flowchart. Sun (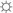) and moon (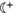) pictograms indicate the light and dark periods, respectively. Solid lines indicate feeding and fasting periods. Experimental schedule from day 0 to day 8: (a) mice entrained to a 12‐h light–dark cycle with ad libitum access to food and water. (b) mice in daytime feeding groups. (c) mice in nighttime feeding groups. (d) mice were killed on day 8 at noon (HALO 05) and midnight (HALO 17), and kidney specimens and serum samples were collected. Sacrification for histopathological analysis was performed at HALO 05. White arrows indicate the time of pioglitazone administration. HALO, hour after light onset.

BALB/c male mice were housed individually in single cages to avoid aggression on a 12:12 light–dark cycle (lights on at 7 am, lights off at 7 pm), with food and water available ad libitum. The study was approved by the Ethics Committee of Poltava State Medical University. Eight‐week‐old mice were randomly assigned to daytime feeding and nighttime feeding groups. Water was available ad libitum, whereas mice in daytime feeding groups received food from 7 am to 7 pm, and in nighttime feeding groups from 7 pm to 7 am (Xin et al., 2021). PG was administered in a dose of 20 mg/kg per os as an aqueous suspension of 40 μl at 7 am or 7 pm as described in our previous study (Fedchenko et al., 2022). Each group consisted of 12 animals. All manipulations at the dark phase were provided under red light. Figure 1 shows the study design.

On day 8 of the feeding intervention, mice were killed by cervical dislocation at noon (05 h after light onset [HALO]) and midnight (HALO 17). The kidneys were removed, immediately frozen, and kept at −80°C until use. Urine samples were collected at 12‐h intervals on the day before the sacrification.

### 2.2 RNA preparation and quantitative reverse transcription PCR

Six mice at each of two timepoints (HALO 05 and HALO 17) were used for RNA extraction. Kidney outer medulla samples were collected. Total RNA was extracted from the kidney using RNeasy kit (QIAGEN), Cat. No. 74104.

To generate single‐strand DNAs, total RNA (≈1 μg) was reverse transcribed using QuantiTect®Reverse Transcription Kit (QIAGEN), Cat. No. 205313.

For SYBR Green‐based analysis, the cDNA equivalent of 50 ng of total RNA from each sample was amplified in the CFX96TM Real‐Time PCR Detection System (BIO‐RAD) using a QuantiTect®SYBR‐Green PCR Kit (QIAGEN), Cat. No. 204143.

Each sample was analyzed in duplication to ensure the accuracy of the data. The gene expressions were detectable as 2^−ΔCT^. All the values were normalized to the expression of the housekeeping gene β‐actin which had weak circadian variation in kidney (Matsumura et al., 2014).

The sequences of specific primers used for real‐time PCR are provided in Table 1. All oligonucleotides were purchased from Metabion International AG.

**TABLE 1:** Primer sequences for mRNA measurement

Gene	Primer sequences	References	ID number
*Arntl*	Forward ACATAGGACACCTCGCAGAA Reverse AACCATCGACTTCGTAGCGT	Liu et al. (2007)	211116B056A01 211116B056B01
*Clock*	Forward CCTATCCTACCTTCGCCACACA Reverse TCCCGTGGAGCAACCTAGAT		211116B056C01 211116B056D01
*Nr1d1*	Forward CGTTCGCATCAATCGCAACC Reverse GATGTGGAGTAGGTGAGGTC		211116B056A03 211116B056B03
*Cry1*	Forward TTGCCTGTTTCCTGACTCGT Reverse GACAGCCACATCCAACTTCC		211116B056C03 211116B056D03
*Cry2*	Forward TCGGCTCAACATTGAACGAA Reverse GGGCCACTGGATAGTGCTCT		211116B056E03 211116B056F03
*Per1*	Forward CATGACTGCACTTCGGGAGC Reverse CTTGACACAGGCCAGAGCGTA		211116B056G03 211116B056H03
*Per2*	Forward GGCTTCACCATGCCTGTTGT Reverse GGAGTTATTTCGGAGGCAAGTGT		211116B056A04 211116B056B04
*Nfe2l2*	Forward CGCCGCCTCACCTCTGCTGCCAGTAG Reverse AGCTCATAATCCTTCTGTCG	Ghosh et al. (2017)	211116B056C05 211116B056D05
*Pparg*	Forward CCAGAGCATGGTGCCTTCGCT Reverse CAGCAACCATTGGGTCAGCTC	Illesca et al. (2019)	211116B056G04 211116B056H04
*Rela*	Forward GAGGTCTCTGGGGGTACCAT Reverse AAGGCTGCCTGGATCACTTC		211116B056A05 211116B056B05
*Scnn1g*	Forward ATGCTTCCAAACGAAGATGG Reverse AGTTGGGGTGTTGCTGGTAG	Borsting et al. (2012)	Purchased “Ukrainian Genetic Technologies LTD”
*β‐Actin*	Forward ACTGCCGCATCCTCTTCCTC Reverse CTCCTGCTTGCTGATCCACATC	Illesca et al. (2019)	211116B056E01 211116B056A02

### 2.3 Urine measurement

For urine collection, mice were put in metabolic cages. K^+^ and Na^+^ were measured in urine by flame photometry. Urine volume, food, and H_2_O intake were determined by the gravimetric method at dark (7 pm–7 am) and light (7 am–7 pm) periods.

### 2.4 Histological analysis

The renal structure was examined by histopathology on paraffin‐embedded kidney tissue sections 4 μm stained with Hematoxylin and Eosin (H&E). Morphological analyses were performed using a light microscope Axio Lab.A1 (Carl Zeiss) and Zen 2.5 lite (blue edition) software. All biopsies were reviewed by the same pathologist, blinded to experimental group affiliation of biopsies.

The histological checklist was used as described (Hesketh et al., 2014; Lamby et al., 2020; Martínez‐Girón et al., 2018). Infiltration foci were assessed at 200× magnification and also were taken into account using the binary system. Blood stasis is recognized in the tissue samples presented when only one or a few blood vessels were more or less filled with erythrocytes, and the majority of blood vessels were devoid of erythrocytes. On the basis of cellular integrity and morphology, we marked and counted tubules as either viable (intact cell morphology) or injured (compromised cell integrity, abnormal cell morphology, and/or loss of cells). The distal tubules were discriminated by cuboidal epithelial cells without brush border morphology of their walls, and with relatively wide lumens (Scudamore, 2014). In case of uncertainty in the identification of distal tubules, they were not taken into account. We captured five images at 400× magnification within the outer medulla for each section to count the mean and expressed the number of injured tubules as a proportion of the total number of tubules.

### 2.5 Statistical analysis

Statistical analysis was performed using GraphPad Prism 5.0 (GraphPad Software) by means of descriptive statistics (M ± SD). Gene expression and urinary excretion data were compared by two‐way ANOVA and post hoc Bonferroni test. For histopathological data 2 × 2 table statistic, including Fisher's exact test and Chi‐square test, was used to compare proportions. *p* < 0.05were considered statistically significant in all the analyses.

The null hypothesis tested was that reverse feeding, PG, and the time of its administration have no influences on the clock and metabolism‐related gene expression in the kidney inner medulla impaired by reversed feeding.

## 3 RESULTS

### 3.1 Effects of feeding intervention and PG intake on diurnal clock gene expression in mouse kidney

**FIGURE 2:**
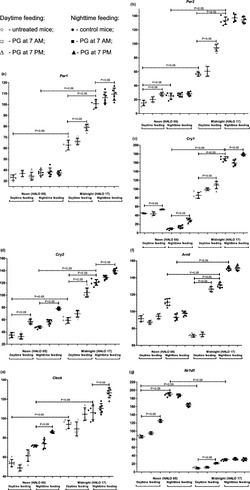
Circadian changes in mouse clock gene mRNA transcription in kidney tissues. Expression of mRNA: (a) *Per1*; (b) *Per2*; (c) *Cry1*; (d) *Cry2*; (e) *clock*; (f) *Arntl*; (g) *Nr1d1*. Significant differences are shown by the horizontal line (*p* < 0.05). Y axis—Relative mRNA levels

Figure 2 shows the expression pattern of clock genes in mouse kidney on the eighth day after initiation of the feeding intervention. In the nighttime feeding group, expression of *Per1, Per2, Cry1, Cry2, Clock*, and *Arntl* was statistically significantly higher at midnight (HALO 17) than at noon (HALO 05) (*p* < 0.05). On the other hand, the expression of *Nr1d1* was higher at noon. Administration of PG at 7 am did not influence the clock gene expression. PG intake at 7 pm slightly increased the *Per1, Cry1, Cry2*, and *Clock* expression at midnight, and the *Cry1, Cry2*, and *Clock* expression at noon (*p* < 0.05).

The daytime feeding led to significant changes in gene expression. Expression of *Per1, Per2, Cry1, Cry2*, and *Arntl* had a decreased level at midnight in comparison with nighttime feeding mice (*p* < 0.05). In addition, we observed a decrease in *Per2* and *Nr1d1* expression at noon in comparison with the nighttime feeding group. PG intake at 7 am did not reveal an influence on the expression of core clock genes at all time points in daytime feeding mice. In contrast, administration of PG at 7 pm induced an increase in *Per1, Per2, Cry1, Cry2, Arntl*, and *Nr1d1* expression at midnight as well as an increase in *Per2, Cry1, Cry2*, and *Nr1d1* expression at noon (*p* < 0.05).

### 3.2 Effect of feeding intervention and PG intake on diurnal inflammation‐ and metabolism‐related gene expression in mouse kidney

**FIGURE 3:**
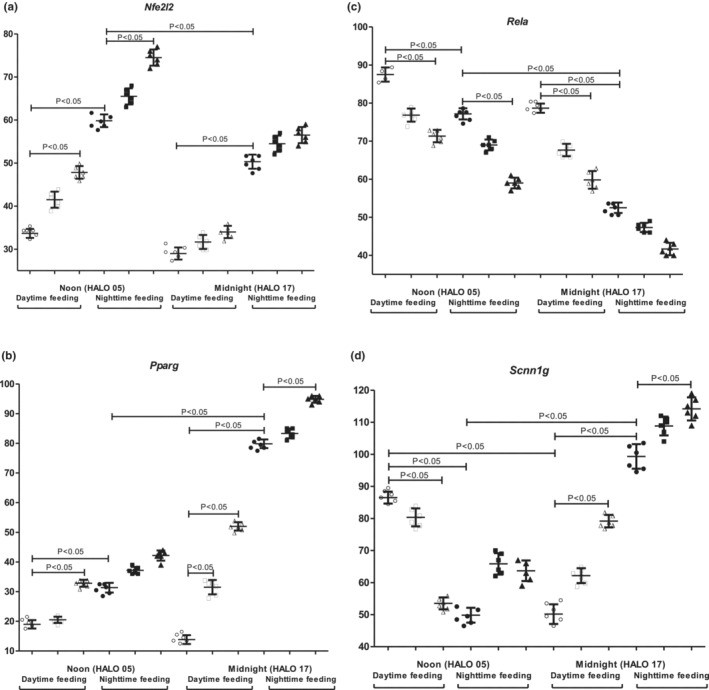
Circadian changes of mRNA transcription of inflammation/metabolism‐related genes in mouse kidney tissues. Expression of mRNA: (a) *Nfe2l2*; (b) *Pparg*; (c) *Rela*; (d) *Scnn1g*. Significant differences are shown by the horizontal line (*p* < 0.05). Y axis—Relative mRNA levels.

Figure 3 shows the expression pattern of inflammation/metabolism‐related genes in mouse kidney after feeding intervention.

In the nighttime feeding group, the expression of *Nfe2l2* and *Rela* was significantly lower at midnight than at noon, in contrast, the expression of *Pparg* and *Scnn1g* was higher at midnight (*p* < 0.05). PG administration at 7 am to nighttime feeding mice did not influence gene expression statistically significantly. We received the data that PG intake at 7 pm increased *Pparg* and *Scnn1g* expression at midnight, as well as *Nfe2l2* at noon. At the same time, we observed a decrease in *Rela* expression after 7 pm PG intake at noon (*p* < 0.05).

The *daytime feeding induced statistically significant differences in gene expression. There was a statistically significant decrease in Nfe2l2*, *Pparg*, and *Scnn1g* expression at midnight with an increase in *Rela* expression. At noon, we observed a decrease in *Nfe2l2*, *Pparg*, and an increase in *Rela* and *Scnn1g* expression in comparison with nighttime feeding animals (*p* < 0.05). PG administration at 7 am to the *daytime feeding* group led to an increase in *Pparg* expression at midnight. PG intake at 7 pm led to an increase in *Pparg*, and *Scnn1g* expression and a decrease in *Rela* expression at midnight. The administration of PG at 7 pm induced an increase in *Nfe2l2*, *Pparg* expression, and a decrease in *Rela* and *Scnn1g* at noon.

### 3.3 Effect of feeding intervention and PG intake on mouse urine metabolism

**FIGURE 4:**
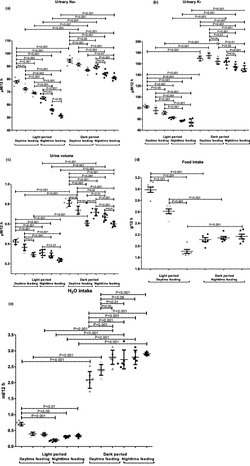
Summary of the effect of feeding intervention and pioglitazone intake on mouse urine metabolism in rest or active periods: (a) urinary Na^+^ concentration; (b) urinary K^+^ concentration; (c) urine volume; (d) food intake; (e) H_2_O intake. Mice had ad libitum access to water. In nighttime feeding groups, mice had access to food in dark period (7 pm–7 am) and were fasted in light (7 am–7 pm) period. In daytime feeding groups, mice had access to food in light period and were fasted in dark period. Urine volume, food, and H_2_O intake were determined at 12‐h dark and light periods. Open and solid dots indicate the daytime and nighttime feeding, respectively. *p*‐value was calculated by two‐way anova followed by Bonferroni post hoc tests

Figure 4 displays the effect of feeding intervention and PG intake on mouse urine production. In the nighttime feeding group, the dark (active) period was characterized by higher levels of sodium, potassium, food, and water intake, as well as urine volume in comparison with the light (inactive) period. PG administration at 7 am to these mice led to a decrease in urinary Na^+^ and K^+^ levels during both dark and light periods. PG intake at 7 pm induced a further decrease in urinary sodium and potassium levels, and urine volume in comparison with control mice as well as mice treated with PG at 7 am.

The *daytime feeding induced a statistically* significant increase in urinary sodium and potassium excretion, urine volume, and food intake during the dark and light periods as well as water intake during light period. PG administration at 7 am decreased urine volume and increased water intake (*p* < 0.01) in the dark period as well as food intake during light period. In these animals, we observed a decrease in urinary Na^+^ and K^+^ levels, urine volume, and water intake during the light period. We received important data that PG intake at 7 pm decreased urinary Na^+^ and K^+^ excretions, urine volume (*p* < 0.05), and increased water intake during the dark period in comparison with control mice as well as food intake during light period. We observed a statistically significant decrease in urine volume only as compared to data after 7 am PG intake. During the light period, more expressive changes took place—a decrease in urinary Na^+^ and K^+^ excretion (*p* < 0.05), as well as urine volume (*p* < 0.05).

### 3.4 Effect of feeding intervention and PG intake on mouse kidney histology

Table 2 summarizes the effect of feeding intervention and PG intake on mouse kidney histology.

**TABLE 2:** The histopathological data and scores of kidney tissue of experimental animals

Histological checklist: Absolute cases per group (percentage)	Time of feeding					
	Daytime feeding			Nighttime feeding		
	Untreated mice (*n* = 13)	PG at 7 AM (*n* = 10)	PG at 7 PM (*n* = 10)	Control mice (*n* = 9)	PG at 7 AM (*n* = 9)	PG at 7 PM (*n* = 10)
Hyperemia in glomerular capillaries	6 (46.2%)	5 (50%)	2 (20%)	1 (11.1%)	0 (0%) *P*_2_ = 0.046 *P*_3_ = 0.033	0 (0%) *P*_2_ = 0.019 *P*_3_ = 0.033
Infiltration foci of kidney interstitium	13 (100%) *P*_1_ = 0.005	8 (80%)	10 (100%) *P*_1_ = 0.011	4 (44.4%)	9 (100%) *P*_1_ = 0.029	9 (90%)
Erythrocytes in the intertubular space	12 (92.3%) *P*_1_ = 0.023	6 (60%)	4 (40%) *P*_2_ = 0.019	4 (44.4%)	2 (22.2%) *P*_2_ = 0.002	0 (0%) *P*_1_ = 0.033 *P*_2_ < 0.0001 *P*_3_ = 0.011
Blood stasis in vessels	13 (100%) *P*_1_ = 0.005	8 (80%)	7 (70%)	4 (44.4%)	8 (88.9%)	4 (40%) *P*_2_ = 0.0021

*Note*: *p*‐value calculated by Fisher's exact test: *P*_1_—when compared with control mice; *P*_2_—when compared with untreated mice; *P*_3_—when compared with PG at 7 am (Daytime feeding).

Reversed feeding led to statistically significant changes in kidney functional morphology such as an increase in mononuclear infiltration foci in the interstitium (*p* = 0.005), and erythrocytes in the intertubular space (*p* = 0.023), and blood stasis in vessels (*p* = 0.005).

PG intake at 7 am did not influence the kidney functional morphology of reversed feeding mice. In contrast, administration of PG at 7 pm decreased the number of animals with erythrocytes in the intertubular spaces (*p* = 0.019). In nighttime feeding mice, PG did not reveal statistically significant changes in kidney morphology.

**FIGURE 5:**
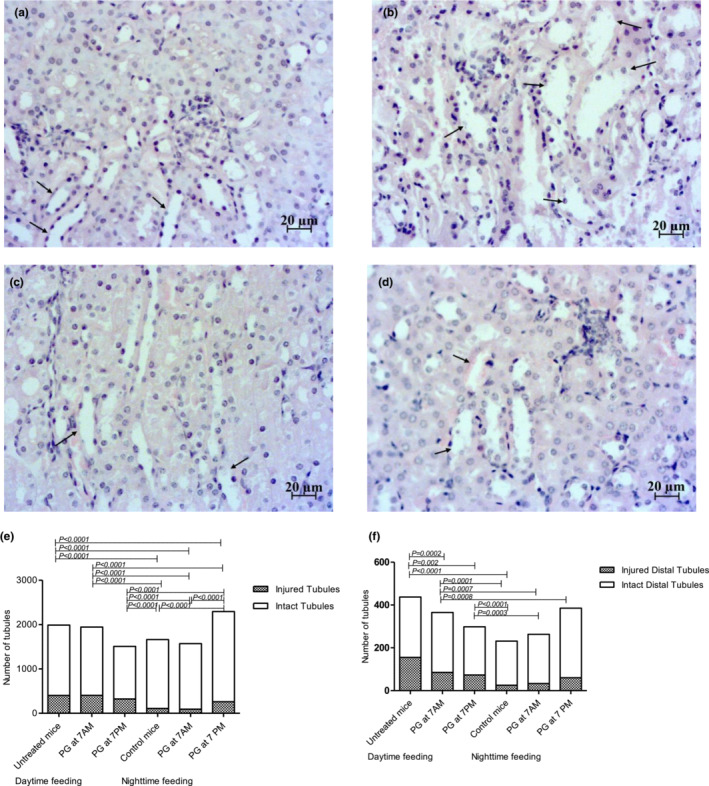
Representative morphology (H&E) and comparisons of proportions of injured tubules of kidney: (a) the distal tubules (arrows) in *control* mice are mostly intact; (b) the majority of distal tubules are injured (arrows) in *untreated* mice; (c) decrease in injured distal tubules (arrows) in daytime feeding *PG* 7 am mice; (d) decrease in injured distal tubules (arrows) in daytime feeding *PG* 7 pm mice; (e) comparisons of proportions of total injured tubules; f, comparisons of proportions of the distal injured tubules. *p*‐value was calculated by chi‐square test.

The major histopathological findings in the kidney outer medulla of reversed feeding mice, which were under influence of PG intake, are presented in Figure 5.

As a field of special interest, we investigated tubules morphology. Reverse feeding increased the number of injured tubules (*p* < 0.0001) and especially injured distal tubules (*p* < 0.0001). Administration of PG at 7 am and 7 pm decreased the number of injured distal tubules (*p* = 0.0002 and *p* = 0.002; respectively).

## 4 DISCUSSION

Circadian rhythms exist in all mammalian tissues throughout the body with as many as 40% of the protein‐coding genes displaying an oscillatory expression (Zhang et al., 2014). Mills and Stanbury found that diurnal urinary excretion of water and electrolytes was not significantly affected by the 12‐h cycle of eating, drinking, and sleeping patterns. They hypothesized an intrinsic mechanism for regulating renal function (Mills & Stanbury, 1952). The mechanism of circadian regulation of kidney functions is recently under study.

The pacemaker of the circadian clock is located in the suprachiasmatic nucleus of the brain and is entrained by light (Dibner et al., 2010). It was shown that metabolic cues likely act as an additional “time giver” for peripheral clocks such as those located in the liver and kidney (Solocinski & Gumz, 2015).

While the circadian clock is composed of many genes, the core molecular clock mechanism is comprised of four key circadian genes: *Clock, Arntl, Per1, 2*, and *3*, and *Cry1, 2* (Partch et al., 2014). The molecular circadian clock mediates the regulation of rhythmic physiological function via transcriptional control and post‐transcriptional control of downstream clock target genes, and this regulation occurs in a tissue‐specific manner. *Arntl, Clock, Per1*, and *Cry2* exhibit clear circadian variation in the whole kidney over a 48‐h period (Pizarro et al., 2013). Moreover, several critically important genes related to sodium transport in the kidney are expressed in a circadian pattern such as the sodium/hydrogen exchanger 3 (NHE3), and the sodium–glucose cotransporter 2 (SGLT2), and the epithelial sodium channel alpha subunit (α‐ENAC) (Panda et al., 2002). At the same time, the kidney has the second greatest number of genes that follow a circadian expression pattern after the liver (Zhang et al., 2014). This pattern consists of membrane channels, transporters, transcriptional factors, enzymes, etc. (Gumz, 2016; Richards & Gumz, 2013; Saifur Rohman et al., 2005; Zhang et al., 2015).

Diet, meal frequency, and light patterns influence rodent peripheral circadian clock expressions (Kuroda et al., 2012; Oishi et al., 2012; Wu et al., 2010). The disruption of the circadian system by irregular or shifted eating leads to the dysregulation of metabolic homeostasis, increased risk of obesity, cardiovascular diseases, impair glucose tolerance, ectopic fat accumulation, diabetes, stroke, and kidney function disorders (El‐Wakil et al., 2007; Michishita et al., 2017, 2016; Patterson & Sears, 2017; Poggiogalle et al., 2018; Wefers et al., 2018). The reversed feeding might impair the temporal expression patterns of proteins that form interlocking transcription–translation feedback loops consisting of seven core clock genes—*Per1, Per2, Cry1, Cry2, Nr1d1*, and *Arntl*.

Our results demonstrate that mice at nighttime feeding had significantly higher kidney *Per1, Per2, Cry1, Cry2, Clock*, and *Arntl* expression at midnight (HALO 17) than at noon (HALO 05). In contrast, the expression of *Nr1d1* decreased at noon. This data went in parallel with other observations (Oishi et al., 2012; Solocinski & Gumz, 2015; Wu et al., 2012, 2010). The reversed feeding influenced the kidney core clock gene expression dramatically: the loss of circadian differences in the expression of *Arntl* and *Cry1* as well as the decrease in *Per1, Per2, Cry1*, and *Cry2* expression at midnight, and the decrease in *Per2, Nr1d1* at noon in comparison with nighttime feeding mice. This data went in parallel with the findings that daytime feeding uncoupled core clock gene expression (Glad et al., 2011; Oyama et al., 2014; Pickel & Sung, 2020) and corresponded to the observations that feeding stimuli induced the desynchronized peripheral circadian rhythm (Wu et al., 2012). *Per1* is the most well‐known core clock gene involved in the rhythmic fluctuation of Na^+^ excretion, aldosterone levels, glomerular filtration rate, and renal blood flow. This clock gene regulates renal *Scnn1a* and other genes responsible for the Na^+^ reabsorption. These include *Per1*—mediated positive regulation of Fxyd5, a positive regulator of Na^+^–K^+^–ATPase, and negative regulation of endothelin ET‐1, a potent inhibitor of ENaC (Richards et al., 2013).

At the same time, we investigated expressions of several inflammation/metabolism‐related genes in mouse kidney tissues—*Nfe2l2, Pparg, Rela*, and *Scnn1g*. Nighttime feeding mice had a decreased expression of *Nfe2l2* and *Rela* at midnight in comparison with the expression at noon. *Pparg* and *Scnn1g* had another pattern with an increased expression at midnight. These expressions correlated with previously described data on the functional connection of *Arntl* and *Nfe2l2* (Early et al., 2018), *Arntl, Clock*, and *Rela* (Magni et al., 2018). A similar pattern of *Scnn1a* expression in mouse kidneys was described by Panda S. et al. (Panda et al., 2002).

The reversed feeding canceled the circadian patterns of *Nfe2l2, Pparg*, and *Rela* expression. Moreover, in these mice, we found a statistically significant decrease in *Nfe2l2* and *Pparg* expression at midnight in comparison with the nighttime feeding group, and an increase in *Rela* and *Scnn1g* expression in kidney tissues. In contrast, at noon, a decrease in *Nfe2l2* and *Pparg* expression and an increase in *Rela* and *Scnn1g* were observed.

Taken together, these findings suppose that reverse feeding uncoupled the peripheral clock gene rhythm in mouse kidney tissues, accompanied by a decreased expression of *Nfe2l2* and *Pparg* as well as an increased expression of *Rela* and *Scnn1g*. These findings correlated with previously described data for liver tissue (Eckel‐Mahan et al., 2013; Fedchenko et al., 2022).

*Nfe2l2* regulates not only circadian clock loops, especially *Cry2* and metabolic signals but is known to be a key factor for anti‐oxidative and anti‐inflammatory properties (Wible et al., 2018; Yamawaki, 2022), mitochondrial homeostasis (Zhuang et al., 2022), H^+^ transport (Bonanno et al., 2022), redox‐sensitivity (Li et al., 2021), and redox‐sensitive potassium channels (Ishii et al., 2013) in kidney tissues. Another transcriptional factor, PPARG, has an important role in the circadian rhythm by counteraction with *Per2* (Bae et al., 2001; Duszka & Wahli, 2020) as well as in kidney function (Corrales et al., 2018). In mice, PPARG targets the *klotho* gene, which is involved in the modulation of urinary phosphate, calcium, and potassium excretion (Izquierdo et al., 2012). PPARG also targets transient receptor potential channel C6 (TRPC6)—a nonspecific calcium‐conducting ion channel (Sonneveld et al., 2017). PPARG tightly interacts with *Nfe2l2* because NRF2 induces *Pparg* expression, and NRF2/PPARG pathway plays an important role in the prevention of kidney injury (Henique et al., 2016; Platt & Coward, 2017). Transcriptional factor NFκB effects are mediated by core circadian protein CLOCK, which can upregulate NFκB‐mediated transcription in the absence of *Arntl*. *Arntl* might counteract the *Clock*‐dependent increase in the activation of NFκB‐responsive genes (Spengler et al., 2012). PPARG and NRF2 antagonize the transcriptional activity of proinflammatory factor NFκB and have protective activity in renal diseases (Guerrero‐Hue et al., 2020; Li et al., 2008; Ruan et al., 2008; Stenvinkel et al., 2021). Little is known about the influence of NFκB on renal electrolyte metabolism. PPARG interacts with γ‐ENAC directly and indirectly and influences Na^+^ reabsorption and K^+^ secretion as well as water uptake (Panchapakesan et al., 2009; Vallon & Lang, 2005).

At the next stage of our research, we studied the effect of feeding intervention on mouse urine metabolism.

The kidney is the organ ultimately responsible for maintaining Na^+^, K^+^, and water balance. In chronic, steady‐state situations, renal excretion of Na^+^, K^+^, and water follows a diurnal rhythm that is independent of the timing of Na^+^, K^+^ intake (Gumz & Rabinowitz, 2013; Koopman et al., 1989; Mills & Stanbury, 1952). The findings suggest that the activity of the renal molecular clock genes serves primarily to conserve Na^+^ (Gumz et al., 2009; Nikolaeva et al., 2016), and has a prominent role beyond *Arntl* (Tokonami et al., 2014).

In our model, the reverse feeding induced a statistically significant increase in urinary sodium and potassium excretion, urine volume as well as food and water intake during the dark and light periods. These changes in water‐electrolyte balance might depend on the decrease in *Per1, Per2, Cry1, Cry2, Arntl*, *Nfe2l2, Pparg*, and *Scnn1g* in mouse kidney tissues. These core clock gene disorders exert a strong influence on renal excretion of Na^+^, K^+^, and water. *Arntl* regulates NHE3 (sodium/hydrogen exchanger 3), OAT3 (organic anion transporter 3) gene expression, *Per1—*NHE3, SGLT1 (sodium–glucose cotransporter 1), and pNCC (phosphorylated sodium chloride cotransporter), ENaC in the kidney tubule (Zhang & Pollock, 2020). Moreover, the loss of PPARG activity might influence the γ‐ENaC activity but this impact is not sufficiently studied (Guan et al., 2005; Nofziger et al., 2005; Vallon et al., 2009). PPARG activation by thiazolidinediones (TZDs) increased Na^+^ reabsorption in the collecting ducts by activating the epithelial sodium channel (Guan et al., 2005; Zhang et al., 2005). On the other hand, there were data that such PPARG activation decreased α‐ENaC and γ‐ENaC in murine cells (Borsting et al., 2012).

Thus, the reversed feeding led to the uncoupling of core clock gene expression accompanied by the disturbances of kidney inflammation‐ and metabolism‐related gene expression, and an increase in urinary sodium, potassium, and water excretion. In parallel, we observed an increase in kidneys' functioning characterized by microcirculation disorders and cell loss, especially in distal tubules (Szeto et al., 2016).

To influence these pathways, we administered PPARG activator PG in a time‐dependent manner at 7 am or 7 pm. The administration of PG at 7 am did not influence core clock genes; in contrast, PG intake at 7 pm statistically significantly increased the lowered expressions of *Per1, Per2, Cry1, Cry2, Arntl*, and *Nr1d1*. PG intake at 7 pm induced a more effective restoration of *Pparg, Nfe2l2*, and *Scnn1g* expression than PG intake at 7 am. We observed important changes in urine components: PG intake at 7 am decreased urine volume and food consumption, as well as Na^+^ and K^+^ excretion; PG administration at 7 pm induced more expressive restoration of urine ion–water composition. At the same time, PG intake normalized kidneys' functional morphology, decreasing the level of microcirculation disorders and the number of injured distal tubules.

Taken together, these results display an ability of PPARG activation by PG to induce core clock gene mRNA expression as well as *Nfe2l2, Pparg*, and *Scnn1g* mRNA expression, stimulating Na^+^ reabsorption and inhibiting K^+^ excretion.

This data support the concept that the PPARG/NRF2/NFκB axis interacts with the peripheral molecular clock (Conway et al., 1995; Fedchenko et al., 2022; Hempel & Köhler, 1965; Ishikawa et al., 1983). PPARG agonist PG enhanced expression of *Scnn1g* mRNA in mouse kidney outer medulla tissues. This enhancement might be mediated through *Per1* (Richards et al., 2013) or directly by PPARG (Guan et al., 2005).

We obtained important data concerning chronopharmacological activity of PG. During reverse feeding (daytime feeding), PG administered at 7 pm had more powerful action in comparison with its intake at 7 am. Thus, such stimulation of PPARG might have an important role in the fine‐tuning of the peripheral molecular clock in the kidney.

The limitation of this study might be the extrarenal effects of PG in this model. We also cannot discriminate the expression of the investigated genes in the different parts of nephrons. The question about direct and indirect stimulation of *Scnn1g* by PPARG is still open. It is early to transfer the obtained results into clinical practice, but clinicians should keep in mind that PG should be taken at the same time daily. The mRNA measurements were provided at two time points and this complicates the interpretation of the 12‐h excretion results. Moreover, other sodium reabsorbing mechanisms besides γ‐ENaC localized along nephron which might be involved in the realization of PPARG effects. The observed urinary electrolytes' changes might reflect either alteration in transport rates in the proximal tubules or being a consequence of a decreased glomerular filtration rate. Thus, further investigation of other sodium transporters as well as glomerular filtration rate are needed. Prospects for future research might be an investigation of core clock inflammation‐ and metabolism‐related genes in the specific nephron compartments such as proximal or distal tubules in reversed feeding mice. Perspective studies might be provided with *Pparg* and/or *Scnn1g* knockout mice as well as in other animal models with the uncoupling of master and peripheral clock gene rhythm. We strongly support the necessity of pilot clinical observation to estimate the chronopharmacological properties of modern TZD in type 2 diabetes patients with normal and impaired kidney function.

## 5 CONCLUSIONS

We suggest that reverse feeding induced disruption of diurnal mouse kidney circadian expression pattern of core clock genes, increasing *Rela* and *Scnn1g* expression, decreasing *Nfe2l2* and *Pparg*, and increasing diurnal urinary Na^+^, K^+^, and water excretion. Administration of PG restores the core clock gene expression pattern, decreases *Rela* and *Scnn1g* expression, increases *Nfe2l2* and *Pparg* mRNA expression, decreases urinary Na^+^ and K^+^ excretion. PG intake at 7 pm was more effective than at 7 am.

## AUTHOR CONTRIBUTIONS

Olga Izmailova: Investigation, methodology, data curation, and statistical analysis. Alina Kabaliei: Investigation and visualization. Viktoriya Shynkevych: Investigation, methodology, visualization, and writing—original draft. Oksana Shlykova: Investigation, methodology, and visualization. Igor Kaidashev: Project administration, methodology, data curation, visualization, writing—original draft, and review and editing.

## FUNDING INFORMATION

The study was a part of research project No. 0120U101166 “The study of the pathogenetic role of the circadian molecular clock in the development of metabolic diseases and systemic inflammation and the development of treatment methods aimed at these processes” funded by the Ministry of Public Health of Ukraine.

## CONFLICT OF INTEREST

All the authors declared no competing interests.

## ETHICS APPROVAL

This study was performed in line with the principles of the Declaration of Helsinki. Ethical approval and consent to participate in this study were approved by the Committee on Bioethics and Ethical Issues of Poltava State Medical University.
